# The mental health and well-being of adolescents with/without intellectual disability in the UK

**DOI:** 10.1017/S204579602300080X

**Published:** 2023-11-30

**Authors:** E. Emerson, V. Totsika, C. Hatton, R. P. Hastings

**Affiliations:** 1 Centre for Disability Research, Faculty of Health and Medicine, Lancaster University, Lancaster, UK; 2 Centre for Research Excellence – Disability and Health, Faculty of Health Sciences, University of Sydney, Sydney, New South Wales, Australia; 3 College of Nursing and Health Sciences, Flinders University, Adelaide, South Australia, Australia; 4 Division of Psychiatry, University College London, London, UK; 5 CEDAR, University of Warwick, Warwick, UK; 6 Tavistock & Portman NHS Foundation Trust, London, UK; 7 Department of Social Care and Social Work, Manchester Metropolitan University, Manchester, UK

**Keywords:** adolescence, inequalities, intellectual disability, mental health, well-being

## Abstract

**Aims:**

To estimate the self-reported and parent-reported mental well-being of adolescents (aged 14 and 17) with/without intellectual disability in a sample of young people representative of the UK population.

**Methods:**

Secondary analysis of data collected in Waves 6 and 7 of the UK’s *Millennium Cohort Study*. The analytic sample consisted of 10,838 adolescent respondents at age 14 (361 with intellectual disability and 10,477 without) and 9,408 adolescent respondents at age 17 (292 with intellectual disability and 9,116 without).

**Results:**

Parental reports of adolescent problems on the Strengths and Difficulties Questionnaire (SDQ) indicated that adolescents with intellectual disability at ages 14 and 17 were more likely to have problems than those without intellectual disability across all SDQ domains. Adolescent self-report data at age 17 indicated that adolescents with intellectual disability were more likely to (self)-report that they had problems than those without intellectual disability on all but one SDQ domain. The magnitude of relative inequality between those with and without intellectual disability was consistently lower for self-report than parental report. On indicators of depression, mental well-being, self-harm, positive mental health, happiness and general psychological distress at ages 14 and 17, we found no self-reported group differences between adolescents with and without intellectual disability.

**Conclusions:**

Further research is needed to understand: (1) why the magnitude of mental health inequalities between those with and without intellectual disability on the SDQ may be dependent on the identity of the informant; and (2) whether such differences are also apparent for other measures of mental health or well-being.

## Introduction

International evidence reviews (Buckley *et al.*, 2020; Einfeld *et al.*, 2011; Totsika *et al.*, 2022) have indicated that children and young people with intellectual disability have poorer mental health and lower well-being when compared to their peers. These inequalities in mental health and well-being appear to emerge in early childhood (Emerson and Einfeld, 2010) and persist across childhood and adolescence.

However, the supporting literature has two notable limitations. First, much of the literature is based on administratively defined convenience samples, with the number of studies based on samples that are representative of national populations of children and young people being extremely limited. For example, a systematic review of the mental health of children and young people with intellectual disability undertaken over the period ending December 2018 identified 19 studies (Buckley *et al.*, 2020). Of these, only nine (47%) were judged to have used ‘appropriate’ sampling frameworks, with only one using a framework that was likely to be representative of a national population (Emerson, 2003). Other sampling frames that were deemed appropriate included country regions (e.g., Einfeld and Tonge, 1996; Taanila *et al.*, 2003) and single cities (e.g., Gillberg *et al.*, 1986; Soltau *et al.*, 2015). Second, most of the data collected on the mental health and well-being of children and young people with intellectual disability are based on third-party (primarily parental) report. Few epidemiological studies reported self-report data from children and young people with intellectual disability themselves. This omission is important on two counts. First, research undertaken on the general population of children and young people indicates that there is only a relatively modest degree of correspondence between parent and child reports of mental health and well-being (Achenbach *et al.*, 2002; Berman *et al.*, 2016; Hemmingsson *et al.*, 2017; Liu *et al.*, 2017). Second, given that this omission is not typically associated with explicit assessment of children’s (in)ability to self-report, it disenfranchises children and young people with intellectual disability from commenting on their own situation.

Secondary analysis of data from nationally representative health and social surveys provides one approach to addressing these issues (e.g., Emerson, 2003; Emerson and Hatton, 2007; Hatton *et al.*, 2018). However, the value of such an approach is dependent on two issues: (1) the ability to identify the intellectual disability status of children and young people within these samples (cf., Emerson *et al.*, 2013); and, in the case of being inclusive of young people’s as well as proxy reporting of mental health, (2) whether children and young people with intellectual disability can provide valid responses to non-adapted measures of well-being and mental health. Very little is known about the latter issue. However, it is possible that a modest proportion of children and young people with intellectual disability can provide valid self-report on some standard and widely used self-report measures of health and well-being. For example, Emerson (2003) used information from parents and teachers to identify 124 11- to 15-year-old children as having intellectual disability in the 1999 Office for National Statistics (ONS) survey of the *Mental Health of Children and Adolescents in Great Britain* (Meltzer *et al.*, 2000). Of these, 79% completed the self-report Strengths and Difficulties Questionnaire (SDQ; Goodman *et al.*, 2000). Analysis of these self-reported responses indicated that: (1) the internal consistency of the SDQ subscales was equivalent for children with/without intellectual disability; (2) there was no evidence of response bias among children with intellectual disability; (3) the pattern of child self-reported difficulties was consistent with independent International Classification of Diseases 10th Revision (ICD-10) diagnoses; and (4) the degree of correspondence between child self-report and parental and teacher reports was modest, a pattern that was equivalent for children with/without intellectual disability.

Our aims were to address the limitations in the existing literature by examining the self-reported and parent-reported mental health and well-being of adolescents (at ages 14 and 17) with/without intellectual disability in a sample representative of the UK population.

## Methods

We conducted a secondary analysis of data collected on 14- and 17-year-old adolescents in Waves 6 and 7 of the UK’s *Millennium Cohort Study* (MCS) (Fitzsimons *et al.*, 2020). The MCS followed a two-stage complex stratified sampling design with oversampling from disadvantaged and ethnic minority areas. Child Benefit Records, a non-means-tested benefit with a near-universal coverage of UK children at the start of the MCS, were used to randomly select participants. Information was collected from parental informants on 11,726 adolescents at age 14 (63% retention from Wave 1), 10,971 (94%) of whom provided self-report data, and from 9,528 adolescent informants at age 17 (51% retention from Wave 1). Data used in the present analyses were collected by computer-assisted personal interview with a parental informant and, separately, computer-assisted self-interview or personal interview with the adolescent. At Wave 6, adolescent respondents were given the option of computer-assisted self-interview or personal interview. Respondents with intellectual disability were significantly more likely than their peers to opt for personal interview (15.2% vs. 0.8%, *p* < 0.001). At Wave 7, two forms of adjustments were available for adolescent respondents: assistance with completion and proxy responding. Both adjustments were more likely to be taken up by respondents with intellectual disability than their peers (assistance 5.1% vs. 0.2%; proxy responding 2.4% vs. 0.4%, *p* < 0.001).

### Measures

#### Intellectual disability

Identification of intellectual disability was primarily based on the results of standardised cognitive assessments undertaken in MCS at ages 3, 5, 7 and 11. At each age, principal components analysis was used to extract a general factor from the results of administered tests. First, we identified children as having potential intellectual disability if they scored more than two standard deviations below the weighted sample mean on the general factor at age 7. If these data were not available, we used data from age 5 and then (if data were still missing) from age 3. This process allowed us to classify potential intellectual disability for 99.1% of children participating at age 7. For 125 children, no cognitive test results were available at any age as interviewers did not administer cognitive assessments under certain circumstances (e.g., if the child ‘has a learning disability … which prevents them from carrying out the assessments’). For these children, we identified intellectual disability based on parental report at age 7 that: (1) the child was reported to be receiving special education due to their ‘learning difficulty’ and (2) the child was reported to have ‘great difficulty’ in reading, writing and maths. Finally, we used the results of cognitive testing at age 11 to reclassify children if their performance was inconsistent with the existing classification (e.g., a child classified as having potential intellectual disability who scored at or above the population mean on a verbal similarities test at age 11). This procedure led to the identification of 647 of the 18,495 (3.5%) children participating at Wave 1 where the child’s mother was the primary informant as having potential intellectual disability, a prevalence rate consistent with the range of estimates from a meta-analysis of epidemiological research (Maulik *et al.*, 2011). As expected, boys were significantly more likely than girls to be identified as having intellectual disability (4.3% vs. 2.6%). Fuller details of this procedure are available in the study by Emerson *et al.* (2019). Data on the presence of intellectual disability were missing for 1.2% of adolescent respondents at age 14 and 1.3% at age 17.

### Mental health and well-being

#### Strengths and Difficulties Questionnaire (SDQ)

The SDQ is a psychometrically robust instrument commonly used in large-scale population surveys to measure emotional and behavioural difficulties in children and young people (Goodman, 1997, 2001; Goodman *et al.*, 2000; Meltzer *et al.*, 2000; Sadler *et al.*, 2018). It contains five subscales. For the purposes of the present study, we used the recommended binary variables of difficulties scoring in the ‘high’ or ‘very high’ range (compared to the ‘low’ or ‘slightly raised’ range) on each subscale and for total scale score (see http://www.sdqinfo.org/ for recommended scoring). The parent-completed SDQ was administered at ages 14 and 17. The adolescent self-completed SDQ was administered at age 17. Parent-completed data were missing for 3.0% of adolescent respondents at age 14 (with intellectual disability 4.4%, without 2.9%, n.s.) and 10.6% at age 17 (with intellectual disability 15.1%, without 10.5%, *p* < 0.05) (please note that all reports of missing data on outcomes or covariates are based on the analytic sample for whom we had valid intellectual disability data. Adolescent-completed data were missing for 1.3% of adolescent respondents at age 17 (with intellectual disability 7.2%, without 1.2%, *p* < 0.001). The high rates of missingness for parent-completed SDQ at age 17 were due to parental non-participation in MCS Wave 7 and the first wave of data collection in which the adolescent cohort member was the primary informant. Previous data suggest that both parent-reported SDQ (Murray *et al.*, 2021) and self-reported SDQ (Emerson, 2005) are valid and reliable for children and young people with intellectual disability – as they are for the general population. Within-sample internal consistency (McDonald’s Omega) of the adolescent-reported SDQ was 0.82 for adolescents with and 0.78 for adolescents without intellectual disability at age 17.

#### Short-Form Moods and Feelings Questionnaire (SF-MFQ)

Administered at age 14, the SF-MFQ is a 13-item adolescent-completed questionnaire designed to screen for depression in children and adolescents (Angold *et al.* 1995). Two studies have suggested using a cut-off of eight or higher for major depression (Angold *et al.* 1995; Thapar and McGuffin, 1998). We used this cut-off to create a binary variable for the risk of depression. Data were missing for 2.8% of adolescent respondents at age 14 (with intellectual disability 21.3%, without 2.2%, *p* < 0.001). Within-sample internal consistency (McDonald’s Omega) of the adolescent-reported SF-MFQ was 0.92 for adolescents with and 0.93 for adolescents without intellectual disability.

#### Kessler (K6)

Administered at age 17, the K6 is a 7-item adolescent-completed questionnaire designed to identify non-specific psychological distress (Kessler *et al.*, 2002, 2003). It consists of six questions about depressive and anxiety symptoms that a person has experienced in the last 30 days. Although other methods exist (Kessler *et al.*, 2010), we adopted the scoring rule used in most studies to classify respondents with K6 scores of 13–24 as having probable serious mental illness and those with scores of 0–12 as probably not having serious mental illness. Data were missing for 1.3% of adolescent respondents at age 17 (with intellectual disability 6.2%, without 1.1%, *p* < 0.001). Within-sample internal consistency (McDonald’s Omega) of the adolescent-reported K6 was 0.84 for adolescents with and 0.86 for adolescents without intellectual disability.

#### Self-Harm

At age 14, adolescents were asked to respond to the following computer-presented question: ‘In the past year have you hurt yourself on purpose in any way?’ (Response options: yes/no). At age 17, self-harm was assessed by one lifetime binary question on attempted suicide (‘Have you ever hurt yourself on purpose in an attempt to end your life?’) and six binary questions related to self-harming acts (including non-suicidal self-harm) undertaken in the previous year (*taken an overdose of tablets, cut or stabbed self, burned self, bruised or pinched self, pulled out your hair, hurt yourself some other way).* Self-harm data were missing for 2.0% of respondents at age 14 (with intellectual disability 17.7%, without 1.4%, *p* < 0.001) and 1.5% of respondents at age 17 (with intellectual disability 6.5%, without 1.4%, *p* < 0.001).

#### Short Warwick-Edinburgh Mental Well-being Scale (SWEMWBS)

Administered at age 17, the SWEMWBS is a well-validated 7-item adolescent-completed questionnaire designed to measure positive mental well-being (McKay and Andretta, 2017; Ringdal *et al.*, 2018). Total raw scores from the short-form measure were converted to metric scores (https://warwick.ac.uk/fac/sci/med/research/platform/wemwbs/using/howto/swemwbs_raw_score_to_metric_score_conversion_table.pdf). Data were missing for 1.8% of adolescent respondents at age 17 (with intellectual disability 9.2%, without 1.6%, *p* < 0.001). Within-sample internal consistency (McDonald’s Omega) of the adolescent-reported SWEMWBS was 0.81 for adolescents with and 0.83 for adolescents without intellectual disability.

#### Short Rosenberg Self-Esteem Questionnaire (SRSEQ)

Administered at ages 14 and 17, the SRSEQ is an abbreviated 5-item adolescent-completed version of the commonly used Rosenberg scale for measuring self-esteem (Robins *et al.*, 2001). Data were missing for 3.1% of adolescent respondents at age 14 (with intellectual disability 22.2%, without 2.5%, *p* < 0.001) and 1.6% at age 17 (with intellectual disability 8.6%, without 1.4%, *p* < 0.001). Within-sample internal consistency (McDonald’s Omega) of the adolescent-reported SRSEQ was 0.86 for adolescents with and 0.91 for adolescents without intellectual disability at age 14 and 0.89 and 0.91, respectively, at age 17.

#### Happiness

At age 14, adolescent respondents were asked to rate their happiness on a 7-point scale (1 = completely happy, 7 = not at all happy) in relation to six domains: schoolwork, the way they look, their family, their friends, their school and life as a whole. Satisfaction with life as a whole is considered an aggregate concept spanning satisfaction across individual domains (van Praag *et al.*, 2003), but evaluation of individual domains provides information about aspects of life that may be important to people in different ways; it is therefore recommended to analyse domains separately (OECD, 2013). Therefore, we created simple binary measures of happiness for each item (positive happy response [score 1–3] vs. unhappy or ambivalent response [score 4–7]. Data were missing for 2.5% of adolescent respondents at age 14 (with intellectual disability 20.8%, without 1.8%, *p* < 0.001).

#### Other variables

Adolescent sex was asked as a simple male/female binary question at earlier stages of the MCS. Detailed information on adolescent ethnicity was converted to a simple binary measure: White British vs. minority ethnic status. Sex and ethnicity data were complete for all informants.

### Approach to analysis

In the main stage of analysis, for binary outcome measures of mental health and well-being, we report prevalence rates (with 95% confidence intervals) along with prevalence rate ratios (estimated by Poisson regression, with respondents without intellectual disability being the reference group) adjusted for respondent sex and ethnicity. For scale outcome measures of mental health and well-being, we report means (with 95% confidence intervals) along with linear regression coefficients (with respondents without intellectual disability being the reference group) adjusted for respondent sex and ethnicity.

All analyses were undertaken in Stata 16 using the survey data routines to adjust standard errors given the clustered nature of the MCS sampling design and inverse probability weights provided with the data to take account of known biases in recruitment and retention. As expected, the use of binary outcome measures violated the assumption of equality of mean and variance, with means always being marginally greater than the variance. Given that this may lead to biases in standard errors, we explored the two available approaches for estimating standard errors available in Stata 16 (the default option of linearised compared with jackknife). The differences in estimates were only marginally different and made no substantive difference to the results. Given this, we use the default (linearised standard errors).

Detailed information on the derivation of the inverse probability weights are provided in MCS documentation (Fitzsimons *et al.*, 2020; Mostafa and Ploubidis, 2017). Briefly, 16 potential predictors of unit non-response were investigated at age 17. Missing data in the predictors were imputed using multiple imputation by chained equations (MICE) to create 50 parallel data sets. Logistic regression models for age 17 non-response conditional on all 16 predictor variables were fitted in each imputed dataset and combined using standard rules. From these models, the probability of unit non-response was predicted for each respondent, with the non-response weight calculated as the inverse of the response probability. Significant predictors included such factors as ethnicity, accommodation type, educational attainment, employment history, number or parents/carers in the household, cohort member cognitive ability at age 5 and whether the cohort member was breastfed.

Complete case analyses were undertaken accompanied by sensitivity analyses in which missing data on the outcome variables were imputed. Following guidance from the Centre for Longitudinal Studies (Silverwood *et al.*, 2020a, 2020b), imputation was undertaken using MICE in Stata to create 50 parallel data sets. It was assumed that variables were missing at random. Variables in the imputation model included all outcome variables and the two covariates (sex and ethnicity), as well as a range of auxiliary variables related to living circumstances and socio-economic position that have been predictive of non-response in MCS and other cohort studies. These included indicators of maternal mental health, household income poverty, material hardship, neighbourhood deprivation and exposure of the cohort member to bullying in previous waves.

The analytic sample consisted of 10,838 adolescent respondents at age 14 (361 with intellectual disability, 10,477 without) and 9,408 adolescent respondents at age 17 (292 with intellectual disability, 9,116 without). Attrition from age 14 to 17 was 19% for adolescents with intellectual disability and 13% for adolescents without intellectual disability.

## Results

Prevalence rates (for binary outcomes) or mean scale scores (for continuous outcomes) for respondents with/without intellectual disability and associated regression coefficients adjusted for sex and ethnicity are presented in Table 1 (complete case analysis) and Table 2 (sensitivity analysis involving imputation of missing outcome data). Parent-completed SDQ scores at ages 14 and 17 show marked and statistically significantly poorer mental health among young people with intellectual disability when compared with their peers on all subscales and total difficulties.

**Table 1.** Self- and parental-completed evaluations of mental health and well-being of adolescents with/without intellectual disability (complete case analysis)

	Adolescent			Parent		
Informant	With ID	No ID	APRR/ARC	With ID	No ID	APRR/ARC
Age 14						
SDQ: Emotional				30.9% (25.0−37.6)	14.2% (13.3−15.1)	2.27*** (1.84−2.80)
SDQ: Conduct				27.4% (22.1−33.3)	12.1% (11.1−13.3)	2.20*** (1.74−2.78)
SDQ: Hyperactivity				20.6% (15.2−27.3)	6.9% (6.2−7.7)	2.84*** (2.12−3.81)
SDQ: Peer problems				37.4% (31.3−43.8)	16.9% (15.9−17.9)	2.14*** (1.80−2.54)
SDQ: Prosocial				32.4% (26.8−38.5)	18.1% (17.0−19.2)	1.70*** (1.42−2.03)
SDQ: Total				32.3% (27.6−38.5)	11.2% (10.3−12.1)	2.81*** (2.32−3.39)
SF-MFQ	28.8% (21.5−37.4)	28.7% (27.4−30.0)	1.15 (0.87−1.53)			
SRSEQ (mean)	9.2 (8.7−9.7)	9.5 (9.4−9.6)	0.07 (−0.43−0.57)			
Self-Harm	13.8% (9.5−19.8)	15.5% (14.5−16.5)	1.09 (0.74−1.59)			
Happiness						
Schoolwork	60.1% (51.4−68.2)	72.7% (71.5−73.9)	0.82** (0.71−0.94)			
Appearance	62.2% (54.3−69.5)	60.5% (59.2−61.8)	0.95 (0.84−1.08)			
Family	84.7% (78.7−89.3)	85.2% (84.2−86.1)	0.99 (0.93−1.05)			
Friends	83.1% (77.1−87.8)	87.3% (86.4−88.1)	0.94 (0.89−1.01)			
School	71.9% (65.2−77.7)	75.4% (74.1−76.6)	0.94 (0.86−1.03)			
Life as a whole	74.2% (66.6−80.6)	78.8% (77.7−79.9)	0.92 (0.83−1.01)			
Age 17						
SDQ: Emotional	24.9% (17.7−33.9)	22.5% (20.9−24.1)	1.35* (1.03−1.77)	28.5% (21.6−36.5)	15.3% (14.0−16.7)	2.09*** (1.58−2.74)
SDQ: Conduct	14.6% (8.8−23.3)	5.4% (4.7−6.2)	2.47** (1.42−4.31)	16.9% (11.6−23.8)	7.1% (6.3−8.1)	2.13*** (1.46−3.10)
SDQ: Hyperactivity	15.1% (9.6−23.1)	14.4% (13.1−15.7)	1.09 (0.69−1.73)	14.9% (9.8−22.0)	3.5% (2.9−4.2)	3.73*** (2.34−5.94)
SDQ: Peer problems	28.7% (20.4−38.6)	20.3% (19.0−21.7)	1.43* (1.02−2.01)	36.0% (28.5−44.2)	16.4% (14.9−17.9)	2.18*** (1.74−2.73)
SDQ: Prosocial	20.7% (14.5−28.8)	10.9% (10.0−12.0)	1.60* (1.11−2.30)	27.3% (20.8−35.0)	15.7% (14.4−16.9)	1.64*** (1.24−2.15)
SDQ: Total	19.9% (13.6−28.0)	13.5% (12.4−14.6)	1.60* (1.10−2.32)	29.4% (22.2−37.7)	8.9% (7.8−10.0)	3.25*** (2.39−4.41)
K6	13.1% (8.4−20.0)	15.7% (14.6−16.9)	0.98 (0.63−1.51)			
SWEMWBS (mean/standard error)	16.3 (13.7−18.8)	19.9 (19.5−20.3)	−0.02 (−1.68−1.64)			
SRSEQ (mean/standard error)	9.7 (9.0−10.4)	10.0 (9.9−10.1)	−0.16 (−0.86−0.55)			
Self-Harm						
Attempted suicide (ever)	7.4% (4.1−13.0)	7.8% (7.0−8.7)	1.11 (0.64−1.92)			
In last year ….						
Taken an overdose of tablets	3.2% (1.2−8.1)	2.9% (2.5−3.4)	1.31 (0.49−3.48)			
Cut/stabbed self	8.8% (5.3−14.3)	11.6% (10.6−12.6)	0.91 (0.56−1.50)			
Burned self	10.2% (6.1−16.6)	5.0% (4.2−6.0)	2.21** (1.29−3.78)			
Bruised/pinched self	14.7% (8.7−23.7)	14.9% (13.8−16.1)	1.12 (0.70−1.81)			
Pulled out hair	8.3% (4.7−14.3)	7.3% (6.5−8.1)	1.37 (0.77−2.43)			
Hurt self some other way	3.8% (1.9−7.3)	4.7% (4.0−7.3)	0.88 (0.43−1.79)			

Notes:

* *p* < 0.05,

** *p* < 0.01, and

*** *p* < 0.001.

APRRs are reported for binary indicators of well-being. ARCs are reported for scaled indicators of well-being. Both are adjusted for the sex and ethnicity of cohort members. APRR, adjusted prevalence rate ratio; ARC, adjusted regression unexponentiated coefficient; SDQ, Strengths and Difficulties Questionnaire; SWEMWBS, Short Warwick-Edinburgh Mental Well-being Scale; SF-MFQ, Short-Form Moods and Feelings Questionnaire; SRSEQ, Short Rosenberg Self-Esteem Questionnaire.

**Table 2.** Self- and parental-completed evaluations of mental health and well-being of adolescents with/without intellectual disability (sensitivity analysis with imputed item non-response data for outcomes)

	Adolescent			Parent		
Informant	With ID	No ID	APRR/ARC	With ID	No ID	APRR/ARC
Age 14						
SDQ: Emotional				30.1% (23.9−36.3)	14.2% (13.2−15.1)	2.23*** (1.79−2.75)
SDQ: Conduct				26.9% (21.1−32.7)	12.1% (11.0−13.2)	2.16*** (1.70−2.75)
SDQ: Hyperactivity				20.1% (14.1−26.0)	6.9% (6.2−7.6)	2.80*** (2.08−3.74)
SDQ: Peer problems				36.6% (30.2−43.0)	16.9% (15.9−17.9)	2.08*** (1.73−2.51)
SDQ: Prosocial				32.1% (26.1−38.2)	18.1% (17.0−19.2)	1.68*** (1.39−2.03)
SDQ: Total				31.4% (25.4−37.4)	11.1% (10.3−12.1)	2.72*** (2.25−3.32)
SF-MFQ	34.0% (22.3−45.7)	29.1% (27.7−30.2)	1.30 (0.93−1.80)			
SRSEQ (mean/standard error)	10.0 (9.1−10.9)	9.6 (9.5−9.7)	0.72 (−0.20−1.63)			
Self-Harm	20.0% (7.9−32.1)	15.9% (14.8−17.1)	1.42 (0.80−2.51)			
Happiness						
School work	54.7% (43.8−65.6)	72.0% (70.6−73.4)	0.75** (0.62−0.91)			
Appearance	59.4% (48.5−70.4)	60.4% (58.9−61.9)	0.92 (0.77−1.11)			
Family	78.8% (61.0−96.5)	84.5% (83.0−85.9)	0.92 (0.74−1.15)			
Friends	75.3% (57.8−92.8)	86.5% (85.1−87.9)	0.86 (0.69−1.07)			
School	64.3% (52.6−76.0)	74.7% (73.3−76.0)	0.85 (0.71−1.01)			
Life as a whole	66.5% (55.8−77.3)	78.1% (76.8−79.3)	0.84* (0.71−0.97)			
Age 17						
SDQ: Emotional	24.5% (15.7−33.4)	22.5% (20.8−24.2)	1.35 (0.96−1.88)	27.1% (19.1−35.2)	15.6% (14.1−17.0)	1.97*** (1.45−2.72)
SDQ: Conduct	15.2% (8.0−22.3)	5.8% (4.7−6.9)	2.39** (1.42−4.01)	15.9% (8.9−22.9)	7.5% (6.5−8.5)	1.92** (1.21−3.00)
SDQ: Hyperactivity	18.8% (10.5−27.0)	15.1% (13.6−16.7)	1.26 (0.81−1.95)	14.3% (7.6−21.0)	3.8% (2.9−4.6)	3.35*** (1.95−5.70)
SDQ: Peer problems	31.2% (21.5−40.8)	21.0% (19.3−22.7)	1.52* (1.09−2.10)	34.3% (25.0−43.6)	16.6% (15.1−18.1)	2.05*** (1.54−2.72)
SDQ: Prosocial	22.2% (14.0−30.4)	11.4% (10.3−12.6)	1.63* (1.11−2.39)	26.9% (17.1−36.7)	15.8% (14.4−17.2)	1.60* (1.11−2.32)
SDQ: Total	22.8% (14.3−31.3)	14.1% (12.7−15.5)	1.75** (1.19−2.56)	28.0% (19.5−36.5)	9.2% (7.9−10.4)	3.00*** (2.14−4.26)
K6	15.3% (8.5−22.1)	16.1% (14.6−17.6)	1.11 (0.70−1.79)			
SWEMWBS (mean/standard error)	22.4 (21.0−23.8)	22.5 (22.3−22.7)	−0.43 (−1.82−0.96			
SRSEQ (mean/standard error)	9.9 (9.2−10.6)	10.1 (10.0−10.2)	0.08 (−0.61−0.78)			
Self-Harm						
Attempted suicide (ever)	9.2% (3.1−15.3)	8.2% (6.8−9.7)	1.28 (0.70−2.30)			
In last year ….						
Taken an overdose of tablets	5.1% (0.0−10.5)	3.5% (2.3−4.7)	1.65 (0.68−4.01)			
Cut/stabbed self	10.8% (4.0−17.7)	12.0% (10.4−13.7)	1.06 (0.58−1.93)			
Burned self	11.4% (4.4−18.4)	5.6% (4.2−7.0)	2.20** (1.23−3.94)			
Bruised/pinched self	15.1% (7.4−22.9)	15.4% (13.7−17.1)	1.12 (0.69−1.82)			
Pulled out hair	9.4% (3.3−15.6)	7.7% (6.3−9.2)	1.45 (0.76−2.75)			
Hurt self some other way	5.7% (0.7−10.6)	5.3% (4.0−6.7)	1.12 (0.48−2.61)			

Notes:

* *p* < 0.05,

** *p* < 0.01, and

*** *p* < 0.001.

APRR are reported for binary indicators of well-being. ARC are reported for scaled indicators of well-being. Both are adjusted for the sex and ethnicity of cohort members. APRR, adjusted prevalence rate ratio; ARC, adjusted regression unexponentiated coefficients; SDQ, Strengths and Difficulties Questionnaire; SWEMWBS, Short Warwick-Edinburgh Mental Well-being Scale; SF-MFQ, Short-Form Moods and Feelings Questionnaire; SRSEQ, Short Rosenberg Self-Esteem Questionnaire.

On all SDQ subscales and total difficulties (apart from hyperactivity, where there was no difference), respondents with intellectual disability also reported significantly poorer mental health than their peers. However, when compared to parental report, effect sizes were lower for adolescent self-report on all subscales and total difficulties and significantly lower (with non-overlapping 95% confidence intervals) for hyperactivity and total difficulties. In contrast, at age 14, there were no statistically significant differences between adolescent respondents with and without intellectual disability with regard to depression, self-harm or happiness with one exception; respondents with intellectual disability were less happy with their schoolwork than their peers. Similarly, at age 17, there were no statistically significant differences between adolescent respondents with and without intellectual disability with regard to non-specific psychological distress, self-esteem, positive mental well-being and for all but one indicator of self-harm (burning self).

The sensitivity analysis produced very similar patterns when accounting for missing data patterns. The only marked differences were that in the sensitivity analysis: (1) at age 14, respondents with intellectual disability reported significantly lower levels of happiness with life as a whole than their peers; and (2) at age 17, the difference in adolescent-reported emotional difficulties (SDQ) was no longer statistically significant albeit the magnitude of the APRR was identical.

## Discussion

Analysis of parent reports of behavioural and emotional problems on the SDQ replicated findings from individual research studies and systematic reviews and meta-analyses; young people with intellectual disability were more likely to report having problems than young people without intellectual disability. Across all SDQ problem domains (and in relation to lower prosocial behaviour), adolescents with intellectual disability were typically reported to be twice as likely to have SDQ scores in the clinical range when compared to adolescents without intellectual disability. Although the SDQ is a screening tool for mental health problems in children and adolescents, it does correspond well to clinical diagnoses. Sensitivity analyses accounting for missing outcome data did not appreciably change these findings. Thus, based on parent report, adolescents with intellectual disability face marked mental health inequalities.

When self-report SDQ data were analysed, adolescents at age 17 with intellectual disability were also more likely to (self)-report that they had problems than adolescents without intellectual disability on every SDQ domain bar hyperactivity. Again, sensitivity analyses accounting for missing data led to a similar pattern of findings. However, using data reported by young people with intellectual disability, the mental health inequality was smaller than when reported by parents. For example, the adjusted prevalence rate ratios for the SDQ total difficulties clinical range were 3.25 for parent reports and 1.60 for adolescent reports with no overlap of confidence intervals. Normative data for the SDQ for the UK also show lower proportions of the population with scores in the clinical range for the self-report compared to the parent-report version. Thus, a reduced risk of mental health problems when reported by adolescents compared to parents would be expected. However, it is not clear why comparative prevalence self-reported by adolescents with and without intellectual disability would be reduced compared to parent report, given that the calculated statistics are ratio measures.

Moving to consider other indicators and measures of self-reported mental health and well-being at ages 14 and 17, we found effectively no self-reported differences between adolescents with intellectual disability and those without on outcomes as varied as depression, mental well-being, self-harm, positive mental health, happiness and general psychological distress. These findings were sustained in sensitivity analyses accounting for missing data. Analysis of data on these self-reported indicators suggested no mental health inequalities associated with intellectual disability. It should be noted, however, that there were no significant differences between the strength of association between intellectual disability and adolescent reported emotional difficulties at age 17 (as indicated by marked overlaps in the confidence intervals of risk estimates) and depression at age 14 and non-specific psychological distress at age 17.

These findings relating to self-reported mental health indicators and measures other than the SDQ are not unique in the literature. For example, adolescents with/without intellectual disability in another UK national study had similar scores on the General Health Questionnaire 12 (Hatton *et al.*, 2018); and young adults with/without intellectual impairment (1 Standard Deviation [1SD] below the mean on cognitive tests rather than 2SDs more typical of intellectual disability) during the COVID-19 pandemic also reported similar scores on the Kessler 6 (Totsika *et al.*, 2021).

In the current study, parent-reported versions of mental health measures and indicators other than the SDQ were not available. Thus, we do not know if parents had reported on these constructs whether they would also have reported similarly to adolescents with and without intellectual disability. We cannot rule out that our findings are measure or construct dependent. However, it is important to highlight that, even with the SDQ data, the size of the identified mental health inequality for adolescents with intellectual disability was markedly attenuated when reported by young people themselves. Sceptics might question the validity of the self-reports of adolescents with intellectual disability about their mental health and well-being. However, existing data, at least for the SDQ, suggest that these adolescents can validly self-report (Emerson, 2005). Thus, the current findings cannot be dismissed simply based on questions about measurement validity. In future research, a wider range of self-report mental health and well-being measures need to be examined in terms of their validity for adolescents with intellectual disability.

We also argue that it is crucial to value self-reports about mental health and well-being provided by adolescents with intellectual disability. Their perspectives and experiences lead us to question the generality or specificity of a well-established mental health inequality affecting adolescents with intellectual disability. Future research should turn to the question of why adolescents with intellectual disability themselves report similar levels of mental health problems and well-being as other adolescents and why their parents do not share this view or report the inequality as larger. In-depth qualitative research about their perspectives on mental health and well-being may be elucidating. Young people with intellectual disability might emphasise different indicators when they consider their mental health or well-being. It may also be that other social processes are at work – perhaps young people with intellectual disability have a heightened awareness of their parents’ worries about them and their tendency to be protective, and so they downplay their own distress so as not to worry their families further. Young people with intellectual disability may also be painfully aware of how they are perceived as different to other adolescents and are giving voice to a view that they are more similar than we like to think. These are speculative ideas. The main point is that significant future research is needed to understand the perspectives of young people with intellectual disability about their own mental health and well-being and, given the longitudinal nature of the MCS, how these may change over time.

A significant strength of the current study is that it drew on data from a nationally representative study (the MCS). However, there are also associated limitations to this approach. First, the MCS itself may have lost some of its representativeness after the early waves of data collection due to significant sample attrition over the 17 years of the study (to the wave of data used in the current research). Second, where those with more severe intellectual disability were included in earlier waves of the MCS, they may have been more likely to stop taking part by the age 17 data collection wave (when the young person became the main respondent). Thus, there may be differential attrition, especially for young people with more severe intellectual disability reducing the representativeness of the intellectual disability sample by age 17 at least. However, while problems of attrition clearly exist, recent work undertaken by the Centre for Longitudinal Studies indicates that the representativeness of cohort studies can be maintained by adopting a structured data-driven approach to imputing missing data, as undertaken in the current study (Silverwood *et al.*, 2020a, 2020b). Whether such an approach can eliminate bias in particular subgroups (e.g., people with more severe intellectual disability with significant mental health difficulties) is unclear. Third, because the MCS is a population representative study, the majority of children with intellectual disability included in it will be those with mild intellectual disability. Thus, data about the mental health of children and adolescents with more severe intellectual disability may need to be examined in other samples. Finally, the sample size for the intellectual disability sub-sample in the current research was quite small; further research with larger samples may help in particular with the precision of prevalence estimates.

In addition to the general suggestion for more research including in-depth research on mental health and well-being with young people with intellectual disability, there are considerable advantages to secondary data analysis of health and social surveys in the UK and elsewhere to understand the experiences of young people with intellectual disability (also illustrated by the current study). Therefore, research is needed to help maximise the participation of children and adolescents with intellectual disability in mainstream surveys. In particular, researchers could provide more evidence about: (1) the proportion of children and young people with intellectual disability who can provide valid self-report on standard self-report measures of health and well-being; (2) what adjustments to the procedures surrounding data collection can increase participation; and (3) for those who cannot participate with appropriate procedural adjustments, what direct adjustments to the existing standardised measures can increase participation or what bespoke measures may be needed to assess mental health and well-being in young people with intellectual disability (Davison *et al.*, 2022).

## Data Availability

MCS data used in this article are freely available, following authorisation, from the UK Data Service (https://ukdataservice.ac.uk/).
